# Factors predictive of invasive ductal carcinoma in cases preoperatively diagnosed as ductal carcinoma in situ

**DOI:** 10.1186/s12885-020-07001-1

**Published:** 2020-06-03

**Authors:** Koji Takada, Shinichiro Kashiwagi, Yuka Asano, Wataru Goto, Tamami Morisaki, Katsuyuki Takahashi, Hisakazu Fujita, Tsutomu Takashima, Shuhei Tomita, Kosei Hirakawa, Masaichi Ohira

**Affiliations:** 1 Department of Breast and Endocrine Surgery, Osaka City University Graduate School of Medicine, 1-4-3 Asahi-machi, Abeno-ku, Osaka, 545-8585 Japan; 2 Department of Pharmacology, Osaka City University Graduate School of Medicine, 1-4-3 Asahi-machi, Abeno-ku, Osaka, 545-8585 Japan; 3 Department of Scientific and Linguistic Fundamentals of Nursing, Osaka City University Graduate School of Nursing, 1-5-17 Asahi-machi, Abeno-ku, Osaka, 545-0051 Japan; 4 Department of Gastrointestinal Surgery, Osaka City University Graduate School of Medicine, 1-4-3 Asahi-machi, Abeno-ku, Osaka, 545-8585 Japan

**Keywords:** Invasive ductal carcinoma, Ductal carcinoma in situ, Invasion, Platelet-lymphocyte ratio, Biopsy, Surgery

## Abstract

**Background:**

Invasion is often found during postoperative pathological examination of cases diagnosed as ductal carcinoma in situ (DCIS) by histological examinations such as core needle biopsy (CNB) or vacuum-assisted biopsy (VAB). A meta-analysis reported that 25.9% of invasive ductal carcinoma (IDC) cases are preoperatively diagnosed by CNB as DCIS. Risk factors for invasion have been studied by postoperative examination, but no factors have been found that could be obtained preoperatively from blood tests. In this study, we investigated factors predictive of invasion based on preoperative blood tests in patients diagnosed with DCIS by preoperative biopsy.

**Methods:**

In this study, 118 patients who were diagnosed with DCIS by preoperative biopsy were included. Biopsies were performed with 16-gauge CNB or VAB. Peripheral blood was obtained at the time of diagnosis. This study evaluated absolute platelet count, absolute lymphocyte count, lactate dehydrogenase, carcinoembryonic antigen, and cancer antigen 15–3 (CA15–3). The platelet–lymphocyte ratio (PLR) was calculated by dividing the absolute platelet count by the absolute lymphocyte count, and patients were grouped into high PLR (≥160.0) and low PLR (< 160.0) groups.

**Results:**

Invasion was found more frequently after surgery in pathologically high-grade cases than in pathologically not-high-grade cases (*p* = 0.015). The median PLR was 138.9 and 48 patients (40.7%) were classified into the high PLR group. The high PLR group was significantly more likely to have invasion detected by the postoperative pathology than the low PLR group (*p* = 0.018). In multivariate analysis of factors predictive of invasion in postoperative pathology, a high PLR (*p* = 0.006, odds ratio [OR] = 3.526) and biopsy method (VAB vs. CNB, *p* = 0.001, OR = 0.201) was an independent risk factor.

**Conclusions:**

The PLR may be a predictor of invasion in the postoperative pathology for patients diagnosed with DCIS by preoperative biopsy.

## Background

Ductal carcinoma in situ (DCIS) is not an invasive malignant tumor; hence, it does not have the ability to metastasize. Therefore, the necessity of surgical treatment and sentinel lymph node biopsy for DCIS has been studied [1–4]. However, DCIS is diagnosed by histological examinations such as core needle biopsy (CNB) or vacuum-assisted biopsy (VAB), and invasion is often found in the postoperative pathological examination. A meta-analysis reported 25.9% (18.6–37.2%) of invasive ductal carcinomas (IDCs) are preoperatively diagnosed as DCIS by CNB [5]. Although risk factors have been examined, no such factors exist that can be identified easily using blood tests.

Cancer affects the general body condition as it progresses. In particular, changes in the blood composition are often observed starting from an early stage. Tumor markers are often correlated with progression and they have been reported to change following recurrence before other symptoms can be detected using different tests [6–8]. Carcinoembryonic antigen (CEA) and cancer antigen 15–3 (CA15–3) are commonly used as tumor markers for breast cancer. The white blood cell population and blood chemistry can also change. Lactate dehydrogenase (LDH) is one of the most important metabolic enzymes involved in glycolysis [9]. An increase in serum LDH is observed with tissue destruction caused by cancerous growth [10], and serum LDH values ​​have been reported to be consistent with clinical TNM staging [10, 11]. Furthermore, the peripheral blood platelet–lymphocyte ratio (PLR) has been reported to be useful for predicting prognosis [12–14], and results from a meta-analysis suggested a correlation between the PLR and progression in breast cancer [12].

Therefore, we hypothesized that there may be a difference in blood test results if invasion occurs in patients diagnosed with DCIS by preoperative biopsy. In this study, we identified predictors of invasion from preoperative blood tests in patients diagnosed with DCIS by preoperative biopsy.

## Methods

### Patients

In this study, 100 and 18 patients who were diagnosed with DCIS by preoperative biopsy from August 2007 to January 2018 at the Osaka City University Hospital were included. Two breast pathologists jointly performed the pathological diagnosis and examination. The grade of DCIS was based on the World Health Organization classification [15]. The presence of comedonecrosis and intraductal calcification was examined and lymphoid infiltrate was evaluated with reference to previous reports [16, 17]. Patients with multiple breast cancers were excluded, as were patients with a history of cancer regardless of breast cancer. Biopsies were performed by 16-gauge CNB or VAB with ultrasonography at the discretion of the attending physician. All patients underwent mastectomy or breast-conserving surgery. In both preoperative biopsy and postoperative pathological examination, invasion was examined by Hematoxylin-Eosin staining and immunohistochemical staining. Furthermore, the expression of the estrogen receptor (ER), progesterone receptor (PgR), human epidermal growth factor receptor 2 (HER2), and Ki67 was evaluated by immunohistochemical staining in the biopsy tissue. All patients underwent ultrasonography and computed tomography, and 90 patients (76.3%) underwent magnetic resonance imaging. Based on these results, tumor size was measured. None of the patients in this study had a suspected invasive carcinoma detected by imaging. Cases that were suspected of having lymph node metastases in the image were diagnosed as IDC even if they were diagnosed with DCIS by biopsy, and were excluded from this study.

### Blood sample analysis

Peripheral blood was obtained before the biopsy. This study evaluated absolute platelet count, absolute lymphocyte count, LDH, CEA, and CA15–3. Patients in whom any of these variables was not measured were excluded from the study. The number of blood cells was determined using a hemocytometer. Percentages of different cell types were determined using a Coulter LH 750 Hematology Analyzer (Beckman Coulter, Brea, CA, USA). The PLR was calculated from the preoperative blood sample by dividing the absolute platelet count by the absolute lymphocyte count. Based on previous studies, a PLR value of 160.0 was used as the cutoff value to discriminate between a high PLR (≥160.0) and a low PLR (< 160.0) [18]. For LDH, CEA, and CA15–3, each upper limit of normal range (ULN) was set as a cut-off value (LDH: 120–242 IU/L, CEA: ≤5.0 ng/mL, CA15–3: ≤25.0 U/mL).

### Statistical analysis

All statistical analysis was performed with the JMP software package (SAS, Tokyo, Japan). The relationship between each factor was examined using Pearson’s chi-square test. The odds ratio (OR) and 95% confidence interval (CI) were calculated by logistic analysis. Multivariable analysis was performed using the multivariable logistic regression model. Significance was defined as a *p* value of less than 0.05.

## Results

### Clinicopathological features

The clinicopathological features of 118 patients who were diagnosed with DCIS by preoperative biopsy and met the conditions of this study are shown in Table 1. The median age was 51 (range, 30–78) years, and the median tumor diameter was 17.7 mm (range, 3.0–50.0 mm). A breast lump was the most common cause of consultation in 63 patients (53.4%). As for other symptoms for consultation, 13 patients (11.0%) had nipple discharge, one patient (0.8%) had skin tangles, and one patient (0.8%) had discomfort. Six patients with breast lumps and three patients (2.5%) with nipple discharge had pain. Of all patients, 76 (64.4%) were detected as having subjective symptoms, while 42 (35.6%) were asymptomatic. Forty patients (33.9%) were found by breast cancer screening, and two patients (1.7%) were found by CT examination for other diseases. At consultation, a tumor was palpable in 85 patients (72.0%). VAB was selected for 51 patients (43.2%), but 67 patients (56.8%), which is more than half, were diagnosed preoperatively by 16-Gauge CNB. Ninety-six patients (81.4%) had ER-positive tumors, and 81 patients (68.6%) had PgR-positive tumors. Seventeen patients (14.4%) had a score of 3+ for HER2. Ki67 expression was detected in > 14% in 20 patients (16.9%). Twenty preoperative biopsy specimens (16.9%) were pathologically high-grade. The eight patients diagnosed with low grade by biopsy had the diagnosis changed to intermediate grade by postoperative pathological examination, and two patients diagnosed with intermediate grade by biopsy had the diagnosis changed to low grade by postoperative pathological examination. However, in no patient, diagnosis was changed from low or intermediate to high grade or from high to not-high grade by postoperative pathological examination. Comedonecrosis was found in 64 patients (54.2%), and intraductal calcification was found in 19 cases (16.1%). As for lymphoid infiltrate, 35 patients (29.7%) were evaluated as moderate or severe. Forty-eight patients (40.7%) were found to have invasion by postoperative pathological examination.

**Table 1:** Clinicopathological features of 118 cases diagnosed with DCIS by preoperative biopsy

Parameters	Number of patients (*n* = 118) (%)
Age at operation (years old)	51 (30–78)
Symptoms	
Asymptomatic / Symptomatic	42 (35.6%) / 76 (64.4%)
Palpability	
Impalpabe / Palpable	33 (28.0%) / 85 (72.0%)
Tumor size (mm)	17.7 (3.0–50.0)
Biopsy device	
Core needle biopsy / Vacuum-assisted biopsy	67 (56.8%) / 51 (43.2%)
Estrogen receptor	
Negative / Positive	22 (18.6%) / 96 (81.4%)
Progesterone receptor	
Negative / Positive	37 (31.4%) / 81 (68.6%)
HER2	
≤ 2 / 3	101 (85.6%) / 17 (14.4%)
Ki67	
≤ 14% / > 14%	98 (83.1%) / 20 (16.9%)
Grade of DCIS	
Low, intermediate / High	98 (83.1%) / 20 (16.9%)
Comedonecrosis	
Absence / Presence	54 (45.8%) / 64 (54.2%)
Intraductal calcification	
Absence / Presence	99 (83.9%) / 19 (16.1%)
Lymphoid infiltrate	
Negative, mild / moderate, severe	83 (70.3%) / 35 (29.7%)
Postoperative pathology	
DCIS only / Invasive ductal carcinoma	70 (59.3%) / 48 (40.7%)
Platelets–lymphocyte ratio	median 138.9 (range, 55.0–292.0)
Low / High	70 (59.3%) / 48 (40.7%)
LDH	median 170 (range, 121–452)
≤ ULN / >ULN	105 (89.0%) / 13 (11.0%)
CEA	median 1.6 (range, < 0.5–12.4)
≤ ULN / >ULN	111 (94.1%) / 7 (5.9%)
CA15–3	median 6.6 (range, < 0.5–40.8)
≤ ULN / >ULN	115 (97.5%) / 3 (2.5%)

*DCIS* Ductal carcinoma in situ, *HER2* Human epidermal growth factor receptor 2, *LDH* Lactate dehydrogenease, *CEA* Carcinoembryonic antigen, *ULN* Upper limit of normal

The median LDH level was 170 IU/L (range, 121–452 IU/L), and it was higher than the ULN in 13 patients (11.0%). The median CEA level was 1.6 ng/mL (range, < 0.5–12.4 ng/mL), and it was higher than the ULN in 7 patients (5.9%). In addition, the median CA15–3 level was 6.6 U/mL (range, < 0.5–40.8 U/mL), and in 3 patients (2.5%) it was higher than the ULN. The median PLR was 138.9 (range, 55.0–292.0), and 48 patients (40.7%) who had a PLR > 160 were assigned to the high PLR group.

### Correlations between clinicopathological features and postoperative pathology

**Fig. 1:**
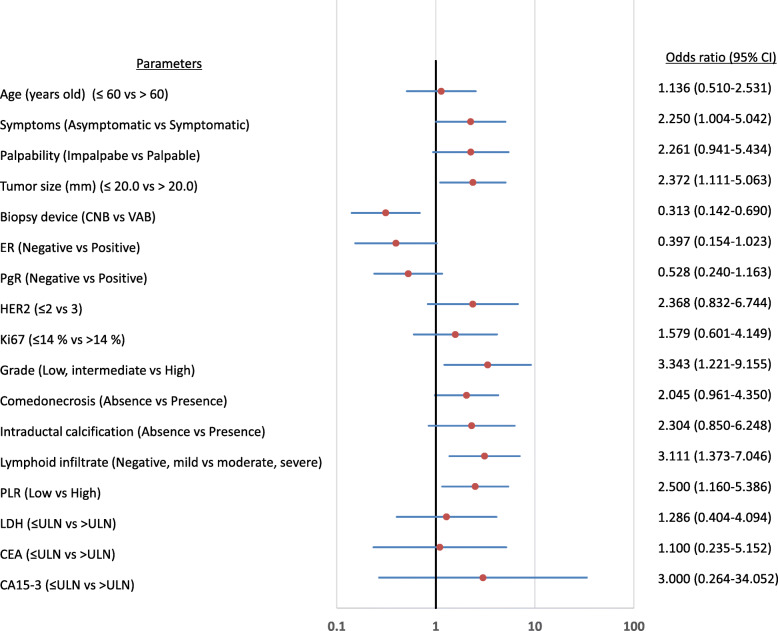
Forrest plot. Forest plot showed odd ratios for the univariate association of the clinicopathological features on postoperative pathology changes to invasive ductal carcinoma. In univariate analysis of factors predictive of invasion in postoperative pathology, a high PLR (*p* = 0.018, OR = 2.500) was a factor, as were larger tumor size (*p* = 0.024, OR = 2.372), non-Low Grade of DCIS (*p* = 0.015, OR = 3.343) and biopsy method (VAB vs. CNB, *p* = 0.003, OR = 0.313)

The correlations between clinicopathological features and postoperative pathology are listed in Table 2. DCIS detected by symptom was significantly more invasive than asymptomatic DCIS (*p* = 0.047). In cases in which the tumor was palpable before surgery, the postoperative pathology tended to be IDC (*p* = 0.065). In cases in which the tumor diameter was larger than 20 mm, the probability of the postoperative pathology being IDC was significantly higher (*p* = 0.024). Cases biopsied by VAB were significantly more likely to be diagnosed as DCIS by postoperative pathology than those biopsied by CNB (*p* = 0.003). Although no significant difference was observed based on immunohistochemical staining, invasion was found more frequently after surgery in pathologically high-grade cases than in pathologically not-high-grade cases (*p* = 0.015). Patients with comedonecrosis and those with intraductal calcification tended to have more invasive disease by postoperative pathology than patients without those conditions (*p* = 0.061, *p* = 0.098, respectively). Invasion rate was significantly higher in patients evaluated as moderate or severe for lymphoid infiltrate than in those evaluated as negative or mild (*p* = 0.018) **(**Fig. 1**)**.

**Table 2:** Correlation between postoperative pathology and clinicopathological features

Parameters	Postoperative pathology		*p* value
	DCIS only (*n* = 70)	Invasive ductal carcinoma (*n* = 48)	
Age at operation (years old)			
≤ 60	50 (71.4%)	33 (68.8%)	
> 60	20 (28.6%)	15 (31.3%)	0.754
Symptoms			
Asymptomatic	30 (42.9%)	12 (25.0%)	
Symptomatic	40 (57.1%)	36 (75.0%)	0.044
Palpability			
Impalpabe	24 (34.3%)	9 (18.8%)	
Palpable	46 (65.7%)	39 (81.3%)	0.065
Tumor size (mm)			
≤ 20.0	48 (68.6%)	23 (47.9%)	
> 20.0	22 (31.4%)	25 (52.1%)	0.024
Biopsy device			
Core needle biopsy	32 (45.7%)	35 (72.9%)	
Vacuum-assisted biopsy	38 (54.3%)	13 (27.1%)	0.003
Estrogen receptor			
Negative	9 (12.9%)	13 (27.1%)	
Positive	61 (87.1%)	35 (72.9%)	0.051
Progesterone receptor			
Negative	18 (25.7%)	19 (39.6%)	
Positive	52 (74.3%)	29 (60.4%)	0.111
HER2			
≤ 2	63 (90.0%)	38 (79.2%)	
3	7 (10.0%)	10 (20.8%)	0.100
Ki67			
≤ 14%	60 (85.7%)	38 (79.2%)	
> 14%	10 (14.3%)	10 (20.8%)	0.352
Grade of DCIS			
Low, intermediate	63 (90.0%)	35 (72.9%)	
High	7 (10.0%)	13 (27.1%)	0.015
Comedonecrosis			
Absence	37 (52.9%)	17 (35.4%)	
Presence	33 (47.1%)	31 (64.6%)	0.061
Intraductal calcification			
Absence	62 (88.6%)	37 (77.1%)	
Presence	8 (11.4%)	11 (22.9%)	0.098
Lymphoid infiltrate			
Negative, mild	56 (80.0%)	27 (56.3%)	
Moderate, severe	14 (20.0%)	21 (43.8%)	0.006
Platelets–lymphocyte ratio			
Low	50 (71.4%)	24 (50.0%)	
High	20 (28.6%)	24 (50.0%)	0.018
LDH			
≤ ULN	63 (90.0%)	42 (87.5%)	
> ULN	7 (10.0%)	6 (12.5%)	0.670
CEA			
≤ ULN	66 (94.3%)	45 (93.8%)	
> ULN	4 (5.7%)	4 (5.7%)	0.904
CA15–3			
≤ ULN	69 (98.6%)	46 (95.8%)	
> ULN	1 (1.4%)	2 (4.2%)	0.353

*DCIS* Ductal carcinoma in situ, *HER2* Human epidermal growth factor receptor 2, *LDH* Lactate dehydrogenease, *ULN* Upper limit of normal, *CEA* Carcinoembryonic antigen

Examination of preoperative blood sampling results showed no significant difference in LDH level or tumor markers based on pre- and postoperative concordance. However, the high PLR group was significantly more likely to show invasion in postoperative pathology than the low PLR group (*p* = 0.018). The correlations between the PLR and other clinical factors were examined, but there was no clear correlation **(**Table 3**)**. In the univariate analysis of factors predictive of invasion in postoperative pathology, a high PLR (*p* = 0.018, OR = 2.500) was a factor, as were larger tumor size (*p* = 0.024, OR = 2.372), high grade of DCIS (*p* = 0.015, OR = 3.343), moderate or severe for lymphoid infiltrate (*p* = 0.006, OR = 3.111), and biopsy method (VAB vs. CNB, *p* = 0.003, OR = 0.313) **(**Fig. 1**)**. Moreover, in multivariate analysis of factors predictive of invasion in postoperative pathology, a high PLR (*p* = 0.006, OR = 3.526) and biopsy method (VAB vs. CNB, *p* = 0.001, OR = 0.201) were independent factors **(**Table 4**)**.

**Table 3:** Correlation between platelets–lymphocyte ratio and clinicopathological features

Parameters	Platelets–lymphocyte ratio		*p* value
	Low (*n* = 74)	High (*n* = 44)	
Age at operation (years old)			
≤ 60	51 (68.9%)	32 (72.7%)	
> 60	23 (31.1%)	12 (27.3%)	0.661
Symptoms			
Asymptomatic	29 (39.2%)	13 (29.5%)	
Symptomatic	45 (60.8%)	31 (70.5%)	0.290
Palpability			
Impalpabe	22 (29.7%)	11 (25.0%)	
Palpable	52 (70.3%)	33 (75.0%)	0.580
Tumor size (mm)			
≤ 20.0	45 (60.8%)	26 (59.1%)	
> 20.0	29 (39.2%)	18 (40.9%)	0.854
Biopsy device			
Core needle biopsy	42 (56.8%)	25 (56.8%)	
Vacuum-assisted biopsy	32 (43.2%)	19 (43.2%)	0.995
Estrogen receptor			
Negative	15 (20.3%)	7 (15.9%)	
Positive	59 (79.7%)	37 (84.1%)	0.556
Progesterone receptor			
Negative	25 (33.8%)	12 (27.3%)	
Positive	49 (66.2%)	32 (72.7%)	0.461
HER2			
≤ 2	63 (82.4%)	40 (90.9%)	
3	13 (17.6%)	4 (9.1%)	0.205
Ki67			
≤ 14%	62 (83.8%)	36 (81.8%)	
> 14%	12 (16.2%)	8 (18.2%)	0.783
Grade of DCIS			
Low, intermediate	60 (81.1%)	38 (86.4%)	
High	14 (18.9%)	6 (13.6%)	0.460
Comedonecrosis			
Absence	36 (48.6%)	18 (40.9%)	
Presence	38 (51.4%)	26 (59.1%)	0.666
Intraductal calcification			
Absence	63 (85.1%)	36 (81.8%)	
Presence	11 (14.9%)	8 (18.2%)	0.635
Lymphoid infiltrate			
Negative, mild	49 (66.2%)	34 (77.3%)	
Moderate, severe	25 (33.8%)	10 (22.7%)	0.204
LDH			
≤ ULN	67 (90.5%)	38 (86.4%)	
> ULN	7 (9.5%)	6 (13.6%)	0.484
CEA			
≤ ULN	70 (94.6%)	41 (93.2%)	
> ULN	4 (5.4%)	3 (6.8%)	0.753
CA15–3			
≤ ULN	71 (95.9%)	44 (100.0%)	
> ULN	3 (4.1%)	0 (0.0%)	0.176
Postoperative pathology			
DCIS only	50 (67.6%)	20 (45.5%)	
Invasive ductal carcinoma	24 (32.4%)	24 (54.5%)	0.018

*DCIS* Ductal carcinoma in situ, *HER2* Human epidermal growth factor receptor 2, *LDH* Lactate dehydrogenease, *ULN* Upper limit of normal. *CEA* Carcinoembryonic antigen

**Table 4:** Univariate and multivariate analysis with upstaging preoperatively DCIS to invasive cancer

	Univarite analysis			Multivarite analysis		
Parameters	Odd ratio	95% CI	*p* value	Odd ratio	95% CI	*p* value
Age at operation (years old)						
≤ 60 vs > 60	1.136	0.510–2.531	0.754			
Symptoms						
Asymptomatic vs Symptomatic	2.250	1.004–5.042	0.047	2.226	0.638–8.463	0.217
Palpability						
Impalpabe vs Palpable	2.261	0.941–5.434	0.065	0.865	0.201–3.568	0.842
Tumor size (mm)						
≤ 20.0 vs > 20.0	2.372	1.111–5.063	0.024	2.647	0.908–8.261	0.075
Biopsy device						
CNB vs VAB	0.313	0.142–0.690	0.003	0.201	0.068–0.534	0.001
Estrogen receptor						
Negative vs Positive	0.397	0.154–1.023	0.051	1.008	0.227–4.633	0.991
Progesterone receptor						
Negative vs Positive	0.528	0.240–1.163	0.111			
HER2						
≤ 2 vs 3	2.368	0.832–6.744	0.100	1.739	0.361–8.417	0.484
Ki67						
≤ 14% vs > 14%	1.579	0.601–4.149	0.352			
Grade of DCIS						
Low, intermediate vs High	3.343	1.221–9.155	0.015	2.234	0.526–9.961	0.274
Comedonecrosis						
Absence vs Presence	2.045	0.961–4.350	0.061	0.817	0.303–2.133	0.682
Intraductal calcification						
Absence vs Presence	2.304	0.850–6.248	0.098	2.525	0.748–9.019	0.136
Lymphoid infiltrate						
Negative, mild / moderate, severe	3.111	1.373–7.046	0.006	2.296	0.752–7.215	0.144
Platelets–lymphocyte ratio						
Low vs High	2.500	1.160–5.386	0.018	3.526	1.423–9.258	0.006
LDH						
≤ ULN vs > ULN	1.286	0.404–4.094	0.670			
CEA						
≤ ULN vs > ULN	1.100	0.235–5.152	0.904			
CA15–3						
≤ ULN vs > ULN	3.000	0.264–34.052	0.353			

*DCIS* Ductal carcinoma in situ, *CNB* Core needle biopsy. *VAB* Vacuum-assisted biopsy. *HER2* Human epidermal growth factor receptor 2, *LDH* Lactate dehydrogenease, *ULN* Upper limit of normal, *CEA* Carcinoembryonic antigen, *CI* Confidence intervals

## Discussion

IDC may be misdiagnosed as DCIS by preoperative biopsy. As mentioned above, 25.9% (18.6–37.2%) of cases preoperatively diagnosed as DCIS have been reported to be IDC according to a meta-analysis [5]. However, the ratio of misdiagnosis in this study was 40.7%, higher than that previously reported. This was greatly influenced by the biopsy method. The meta-analysis found that one of the risk factors for underestimation of invasion was sampling by 14-Gauge CNB instead of 11-Gauge CNB. In contrast, for more than half of the cases in our study 16-Gauge CNB was used for biopsy. Therefore, in patients diagnosed with DCIS by VAB, the rate of postoperative invasion detection was 27.1%, in contrast with that found in patients diagnosed by CNB, which was 52.2%. Certainly, the use of VAB causes stronger pain and has higher medical costs than CNB. However, in the future, CNB with a thicker puncture needle or VAB is considered necessary for a more accurate preoperative diagnosis.

In addition to the different rate of postoperative invasion detected in our study, clinicopathological features also differed from those shown in the meta-analysis [5]. According to the meta-analysis, only 8.3% of all cases diagnosed with DCIS by preoperative biopsy were palpable, and 98.3% were detected by breast cancer screening. While, the pathological diagnosis of high grade was 49.4%, accounting for about half of the cases, in this study, 64.4% of patients had symptoms and 72.0% were palpable. The pathological diagnosis of high grade was 16.9%, which was low. This may be due to the low screening rate in Japan. It is reported that the screening rate in Japan is about 40%, lower than in other countries [19]. The number of DCIS detected early that could not be palpated was small; however, as they progressed, the proportion of patients with symptoms increased, and they became palpable. High grade DCIS may have been diagnosed as invasive ductal carcinoma at biopsy because it has already acquired invasiveness. The reason for a low ratio of a score of 3+ for HER2 may be the same. Although the rate of HER2 overexpression in DCIS has been reported to be from 28 to 65% [20], the rate shown in this study was still lower. We considered that the low ratio of high grade DCIS caused this discrepancy because high grade DCIS was reported to be frequently negative for ER and overexpress HER2 [20].

Various factors other than biopsy devices are considered risk factors for underestimation of invasion; high grade, tumor size larger than 20 mm, and palpability have been previously identified as risk factors [5]. One study also reported hormone receptor negativity as a risk factor [21]. There are reports suggesting that comedonecrosis, intraductal calcification, and lymphoid infiltrate are risk factors [16, 17]. Although this study showed differences in the ratio of invasion by postoperative pathology and clinicopathological features from those presented in previous reports, similar results were found regarding risk factors. However, this study focused on preoperative blood test results, and invasion in postoperative pathology was found significantly more frequently in patients with a high PLR than in patients with a low PLR. Platelets and growth factors such as platelet-derived growth factor and transforming growth factor-β are known to promote tumor growth [22–26]. In addition, immunity is involved in the progression of cancer, and lymphocytes play a key role in the host anti-tumor immune function [27]. This study was based on the hypothesis that blood test changes may occur as cancer progresses. LDH and tumor markers showed no significant difference based on pre- and postoperative concordance, but invasion was significantly more likely to be found in the high PLR group than in the low PLR group. Perhaps PLR did not rise because of the invasion, but in an environment with a high PLR, the tumor could easily acquire invasive ability. High proliferative potential and malignancy, such as HER2-positive and high nuclear grade, cause comedonecrosis and intraductal calcification. If invasion appears, the invasive cancer may have caused inflammation in the surrounding interstitium. In recent years, one study has reported changes in the immune microenvironment of tumors in DCIS and IDC. According to this report, immune escape is progressing in the invasion part [28]. In other words, the trigger of invasion requires a deterioration of the immune environment, and PLR may be the indicator for such deterioration. Although the actions of platelets and lymphocytes are generally reported, it is uncertain whether they actually affect DCIS. In future, we need to examine the biological effects of platelets and lymphocytes on DCIS by immunostaining, gene analysis, and protein quantification in vitro.

There are some limitations to this study. First, there were many cases, in which biopsy was performed with 16-Gauge CNB, so the rate of IDC detection after the surgery was higher than that shown in previous reports. Secondly, some clinicopathological features, such as the ratio of palpability or the grade of DCIS, also differed from those shown in previous reports. The usefulness of mammography scores, so-called the Breast Imaging Reporting and Data System (BI-RADS), has also been reported as a predictor [5]. Third, in this study only 65 patients (55.1%) had mammography performed. Finally, since liver diseases and inflammation easily affect the absolute platelet count and lymphocyte count, it is also a limitation that the comorbidities were not included in the study. However, randomized trials are currently underway to investigate the outcomes during follow-up for low-grade DCIS [29, 30]. One strength of this study is that the PLR can be evaluated relatively easily in clinical practice, and changes in DCIS can be found by evaluating the PLR over time. Furthermore, the current trend is that sentinel lymph node biopsy is being omitted in the diagnosis of DCIS [31, 32]. Some studies reported that metastasis to the sentinel lymph node is unlikely to be found by sentinel lymph node biopsy during surgery for DCIS [31, 33]. If the PLR is high, the invasion may be found by postoperative pathological examination. In addition, chemotherapy may be less effective among these patients. Therefore, we believe that sentinel lymph node biopsy may still be needed in patients with high PLR.

## Conclusions

The PLR may be a predictor of invasion in postoperative pathology for patients diagnosed with DCIS by preoperative biopsy.

## Data Availability

The datasets used and/or analyzed during the current study are available from the corresponding author on reasonable request.

## Abbreviations

CA15–3: Cancer antigen 15–3
CEA: Carcinoembryonic antigen
CI: Confidence interval
CNB: Core needle biopsy
DCIS: Ductal carcinoma in situ
ER: Estrogen receptor
HER2: Human epidermal growth factor receptor 2
IDC: Invasive ductal carcinoma
LDH: Lactate dehydrogenase
OR: Odds ratio
PgR: Progesterone receptor
PLR: Platelet–lymphocyte ratio
ULN: Upper limit of normal
VAB: Vacuum-assisted biopsy
